# Retraction Note to: Inotropic therapy for cardiac low output syndrome: comparison of hemodynamic effects of dopamine/dobutamine versus dopamine/dopexamine

**DOI:** 10.1186/s40001-017-0256-y

**Published:** 2017-05-16

**Authors:** N. E. El Mokhtari, A. Arlt, A. Meissner, M. Lins

**Affiliations:** 1 Department of Cardiology, University Hospital Schleswig-Holstein, Campus Kiel, Kiel, Germany; 2 Department of Internal Medicine, University Hospital Schleswig-Holstein, Campus Kiel, Kiel, Germany

## Retraction

This article [1] has been retracted by BioMed Central because it was republished in the same journal [2]. The authors have informed us that one manuscript was submitted and published as two articles. No further information about the history of these articles is available as the articles were published before BioMed Central took over publication of the journal. Dr. El Mokhtari has not responded to our correspondence about this retraction. All other authors support this retraction.
